# Use of aerobic treadmill exercises on nerve regeneration after sciatic nerve injury in spontaneously hypertensive rats

**DOI:** 10.1590/acb370804

**Published:** 2022-10-28

**Authors:** Gustavo Santiago de Lima Figueiredo, Marcela Fernandes, Vinícius Neves Atti, Sandra Gomes Valente, Felipe Roth, Luis Renato Nakachima, João Baptista Gomes dos Santos, Carlos Henrique Fernandes

**Affiliations:** 1 MD. Universidade Federal de São Paulo – Department of Orthopedics and Traumatology – Division of Hand Surgery – Sao Paulo (SP), Brazil.; 2 PhD. Universidade Federal de São Paulo – Department of Orthopedics and Traumatology – Division of Hand Surgery – Sao Paulo (SP), Brazil.

**Keywords:** Nerve fibers, Exercise Test, Innervation, Motor neurons

## Abstract

**Purpose:**

Various postoperative protocols have been proposed to improve outcomes and accelerate nerve regeneration. Recently, the use of physical exercise in a post-surgical neurorraphy procedure has shown good results when started early. We experimentally investigated the hypothesis that post-operative exercise speeds up results and improves clinical and morphologic parameters.

**Methods:**

Isogenic rats were randomly divided into four groups: 1 SHAM; 2 SHAM submitted to the exercise protocol (EP); 3 Grafting of the sciatic nerve; and 4 Grafting of the sciatic nerve associated with the EP. The EP was based on aerobic activities with a treadmill, with a progressive increase in time and intensity during 6 weeks. The results were evaluated by the sciatic functional index (SFI), morphometric and morphologic analysis of nerve distal to the lesion, and the number of spinal cord motor neurons, positive to the marker Fluoro-Gold (FG), captured retrogradely through neurorraphy.

**Results:**

Functional analysis (SFI) did not show a statistical difference between the group grafted with (–50.94) and without exercise (-65.79) after 90 days. The motoneurons count (Spinal cord histology) also showed no diference between these groups (834.5 × 833 respectively). Although functionally there is no difference between these groups, morphometric study showed a greater density (53.62) and larger fibers (7.762) in GRAFT group. When comparing both operated groups with both SHAM groups, all values were much lower.

**Conclusions:**

The experimental model that this aerobic treadmill exercises protocol did not modify nerve regeneration after sciatic nerve injury and repair with nerve graft.

## Introduction

Despite the known ability of peripheral nerves axonal regeneration, the evolution in treatment, technology and post-surgical results after nerve graft are still quite variable^1^. The distance from the lesion to the innervated site, the rate of axonal regeneration, the progressive decline in renervation capacity of the transected nerve and muscle atrophy are known limiting factors in the result after neurorraphy^2^^–^ 5.

Various postoperative protocols have been proposed in order to improve performance and accelerate nerve regeneration using nutritonal suplements^6^^,^ 7, medication^8^^,^ 9, electric stimulation^1^^,^ 10 ^–^ 12, etc.

Recently, the use of physical exercises in postsurgical neurorraphy procedures has been the subject of some studies with good functional outcomes after peripheral nerve injury. The positive results of this intervention were related to increased expression of brain-derived neurotrophic factor (BDNF) protein and its receptors^13^^,^ 14, activating trascription factor 3 (ATF3)^15^ and improve of the AAK/AMPK (cellular energy sensor) activity^16^. Although good results were reported, other studies^11^^,^ 17 disagree with these associations.

This controversy may be related to some differences between the protocols described, including the time of initial intervention in the postoperative period, intensity and frequency^14^.

Taking the hypothesis that physical exercise improves clinical outcomes after neurorraphy of peripheral nerves, this study will evaluate the effectiveness of the use of aerobical treadmill exercise in neural regeneration after nerve repair with graft in the sciatic nerve of rats.

## Methods

This study was conducted with 30 isogenic male rats with a mean weight of 200–250 g, provided by the Experimental Model Center of the Universidade Federal de São Paulo (CEDEME-UNIFESP). The rats were housed at the Collective Vivarium Research Building CEUA number 4 / UNIFESP, where they were maintained on a 12-h light/dark schedule, at a temperature of 21 ± 2 °C. They received free access to water and a standardized diet (Nuvilab-CR1 from Quimtia S/A, Lima, Peru). Animal protocols were conducted with the approval of the Ethical Committee of the University (ECU No. 9126071015), in accordance with national and international guidelines.

The rats were randomly divided into four different groups as follows: Group I (SHAM): Animals submitted to the surgical procedure, with nerve exposure, used as controls (n = 5); Group II (SHAM+PE): Operated sham animals undergoing to physical exercise protocol (n = 5); Group III (GRAFT): Animals subjected to sciatic nerve graft surgery (n = 10); Group IV (GRAFT+PE): Animals submitted to nerve graft surgery undergoing to physical exercise protocol (n = 10).

### Surgical procedures

The animals were anesthetized with a xylazine solution composed of 50 mg/kg ketamine and 10 mg/kg intraperitoneally. A longitudinal skin incision was performed, opening the muscular plane between vastus lateralis and the biceps femoris with sciatic nerve exposure.

In groups I and II, the procedure stopped at this stage and the incision was sutured with monofilament nylon 4.0. In groups III and IV, surgery proceeded under a magnified view with a surgical microscope and microsurgical instruments. The nerve was gently dissected and an 8 mm segment was removed leaving a distal stump of approximately 3 mm before its branching. This nerve segment, now a nerve graft, was then reversed and sutured in the created defect. After surgery the animals were placed in cages with three animals each, water *ad libitum* and immediately treated with analgesics and antiinflamatory pharmaceuticals.

### Physical exercise protocol on treadmill

The animals in groups II and IV were subjected to forced exercise in a collective belt (AVS Project – www.avsprojetos.com.br) for eight animals with progressive training sessions every 10 days.

Before the surgical procedure the animals become familiar with the device for 2 days by being placed on the conveyor belt for 2 min of a warmup speed of 2 m/min, followed by 5-min session at a speed of 5 m/min with grade 0° tilt.

After 3 weeks from the surgical procedure, the animals started the protocol with a 2-min warmup speed of 2 m/min. In the first 2 weeks, the sessions lasted 10 min at a speed of 5 m/min; the third- and fourth-week sessions lasted 15 min at a speed of 10 m/min; in the fifth and sixth week the sessions were 20 min at a speed of 15 m/min. During the weekend the animals remained at rest.

### Sciatic Functional Index

**Figure 1:**
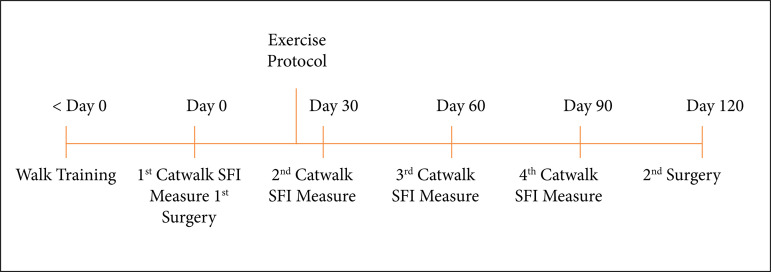
Timeline of interventions.

A walking test was performed before surgery and after 30, 60 and 90 days (Fig. 1). The CATWALK system^18^^–^ 20 was used, which evaluates the animals’ footprint through a transparent walkway coupled to a computer system. This record of the footprint is used to calculate the Sciatic Functional Index (SFI).

### Morphologic and morphometric analysis

After the last measurements on D90, a new procedure was performed to remove sciatic nerve slices. For reasons of material and vivarium in the institution, the procedures were performed in stages. An average of eight rats per week was operated (two from each SHAM group and four from each GRAFT group) to prevent a specific group from being evaluated later than another. A 3-mm nerve segment was removed just distally from the repair site for morphologic and morphometric studies. The nerve segment was fixed and histochemically processed. Semifine slices of 0.5 μm were cut as distal as possible from the lesion on the nerve fragment (2.5–3 mm) in a Reichert–Jung ultramicrotome and stained with toluidine blue for morphologic and morphometric analyses.

The morphometric analysis involved observations of images from the transverse slices of semifine sciatic nerve segments distal to the surgical site. Images were captured with a Zeiss imager MI–AX10 (Zeiss, Jena, Germany) light-transmitting microscope, using a 10 objective and 1 optovar equipped with a JVC TK 1270 video camera (JVC, Wayne, NJ, USA) and transmitted to a computer by picture frame software. After capture of the nerve image, the nerve area and minimum diameter were measured along the internal border of perineurium.

The nerve fibers were identified by a binarization process, in which myelin sheaths appear black and axons appear white. For each myelinated fiber, the following information was recorded: fiber diameter, axonal diameter, myelin sheath thickness, and G ratio (the axonal diameter/ fiber diameter ratio, which influences nerve impulse conduction).

### Fluoro-Gold staining

After the nerve segment resection process (90 to 120 days after surgery), a retrograde tracer, Fluoro-Gold (FG), was used to label motoneurons in the anterior horn of the spinal cord of all animals. Briefly, the right sciatic nerve was exposed to 3% FG for 90 min, after which the muscular layers and skin were sutured to close the incision. After 48 h of exposure to the tracer, the rats were once again anesthetized and perfused through the left ventricle with sequential solutions^21^. Immediately after perfusion with the final solution, a laminectomy was performed, the segment of the spinal cord was removed and the spinal cord segments were cryoprotected and sectioned into frozen slices with a 40-μm thickness.

**Figure 2:**
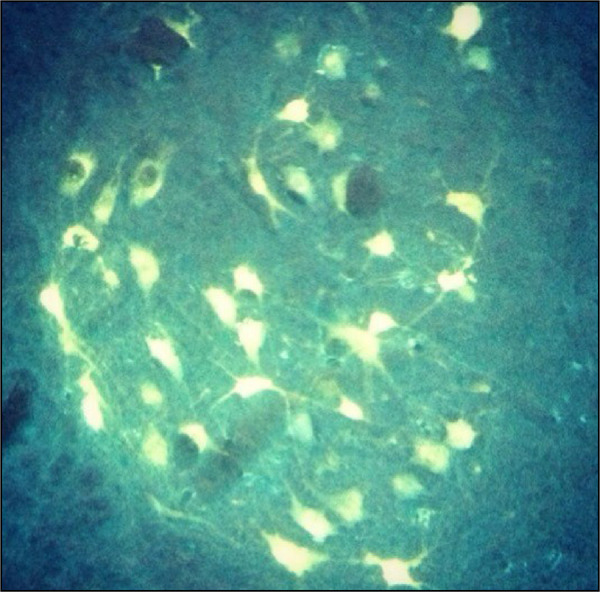
Microscopy of the anterior horn.

The slices were subsequently mounted onto histological glass slides and analyzed by fluorescence microscopy. The number of FG-positive motor neurons in the anterior horn of the spinal cord was recorded. To avoid misinterpretation, only cells strongly positive for FG were included in these measurements. The Abercrombie^22^ correction criteria for serial microtome frozen sections were applied for the total number of cells (Fig. 2).

### Statistical analysis

Differences in the SFI between groups were evaluated by repeated measures analysis of variance (ANOVA). All other comparisons were performed using ANOVA with Bonferroni post-tests. The Kruskal–Wallis test was used for the morphometric analyses when data were not normally distributed. The α-level was set at 0.05.

## Results

During the study, four rats died. Of the 26 remaining animals, 5 were in group I, 5 in group II, 7 in group III and 9 in group IV.

### Sciatic Functional Index

SFI results are described in Table 1. All groups in its first assessment had similar values (SFI0 –16.4, –18.8, –4.4, –10.1, respectively) with no statistical difference between them.

**Table 1:** SFI among the groups across time (0, 30, 60 and 90 days postoperatively).

	Groups	Mean	SD	95% IC	Minimum	Maximum	p-Value
**SFI 0**	SHAM	**–16.39**	11.52	–27.04 to –5.73	–25.74	0.91	p > 0.05
	GRAFT	–4.44	20.80	–26.27 to 17.39	–25.57	24.83	
	GRAFT+PE	–10.07	14.61	–21.30 to –1.16	–35.18	7.45	
	SHAM+PE	–18.83	11.92	–33.63 to –4.03	–30.21	–0.81	
**SFI 30**	SHAM	–6.75	6.62	–12.88 to 0.62	–13.58	4.91	**p = 0.707** GRAFT vs GRAFT+PE; **p < 0.001** SHAM/SHAM+PE vs GRAFT/GRAFT+PE; **p > 0.05** SHAM vs SHAM+PE
	GRAFT	–77.32	8.09	–84.81 to –69.83	–84.33	–62.6	
	GRAFT+PE	–81.45	7.55	–87.26 to –75.64	–96.43	–72.36	
	SHAM+PE	–5.71	8.35	–16.09 to +4.66	–17.34	4.12	
**SFI 60**	SHAM	–14.39	12.56	–26.02 to –2.77	–36.06	1.54	**p = 0.996** GRAFT vs GRAFT+PE; **p < 0.001** SHAM/SHAM+PE vs GRAFT/GRAFT+PE; **p > 0.05** SHAM vs SHAM+PE
	GRAFT	–73.40	7.52	–80.35 to –66.44	–83.1	–63.67	
	GRAFT+PE	–74.57	11.93	–83.75 to –65.39	–91.12	–57.05	
	SHAM+PE	–15.37	8.52	–25.96 to –4.78	–24.18	–3.26	
**SFI 90**	SHAM	–14.97	13.66	–27.61 to –2.34	–38.82	–0.09	**p = 0.734** GRAFT vs GRAFT+PE; **p < 0.001** SHAM/SHAM+PE vs GRAFT/GRAFT+PE; **p > 0.05** SHAM vs SHAM+PE
	GRAFT	–71,12	53.79	–100.69 to –1.18	–79.57	70.56	
	GRAFT+PE	–65.79	11.11	–74.33 to –57.25	–91.73	–55.44	
	SHAM+PE	–10.22	7.164	–19.11 to –1.32	–19.09	0.91	

The SHAM group obtained indices within normal limits in all measurements, with its values remaining always negative and near to zero. As in group SHAM, in group SHAM+PE, the values were very similar and there was no functional difference when comparing the two groups (p = 0.992). In both groups, SFI30 remained constant, and there was degeneration in function after the procedure.

**Figure 3:**
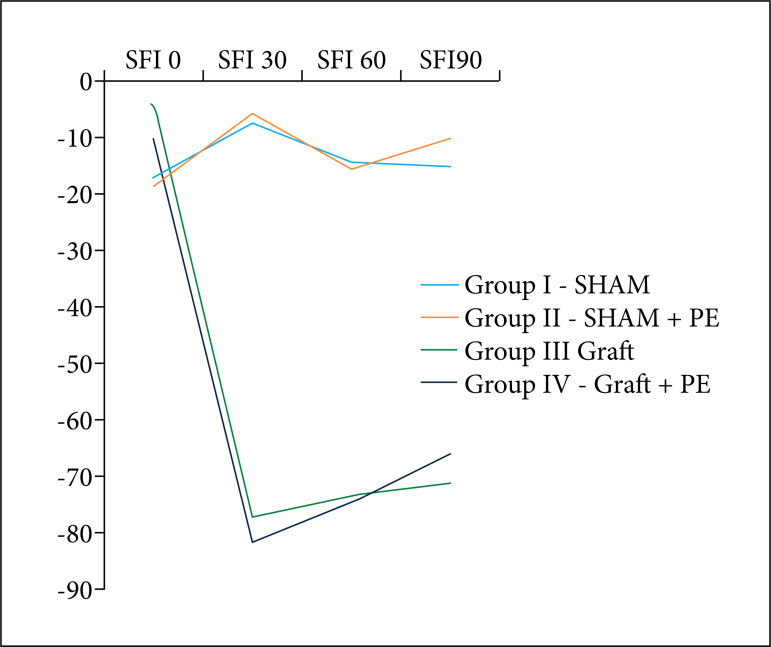
SFI Average at 0, 30, 60 and 90 days.

In the GRAFT and GRAFT+PE groups, as expected, the first measurement after grafting surgery showed a sharp drop in the values of SFI30 (–77.32 ± 8.1 and 7.6 ± –81.45) (Table 1). In the postoperative follow-up, both grafted groups showed a progressive functional improvement with SFI60 values of –73.4 and –74.57, respectively, but still lower than the control groups (Fig. 3). When comparing GRAFT group and GRAFT+PE group, despite the animals submitted to physical exercise presented a greater SFI90 (–65.79, –71.12), there was no statistical significance (p = 0.734).

### Morphological analysis

Nerves in SHAM and SHAM+PE groups had no morphological changes. The fascicles had an unchanged morphology without Wallerian degeneration signals. The nerves of the GRAFT and GRAFT+PE groups presents morphological changes with loss of myelinated fibers of great caliber and a predominance of small-caliber myelinated fibers, suggesting axonal sprouting. Wallerian degeneration presented in both graft groups. All morphological parameters evaluated showed no differences with a statistical significance between the SHAM control group (I) and SHAM+EF group (II).

### Morphometric analysis

All evaluated morphometric parameters showed no differences with a statistical significance between the SHAM control group (I) and SHAM+PE group (II) as well.

The diameter of the fibers was higher in the GRAFT group (7.76 ± 0.27) when compared to the GRAFT+PE group (6.07 ± 0.47) with a statistical significance (p < 0.001). Although higher values were obtained in the GRAFT group, this variable also presents figures about 2/3 of SHAM and SHAM+EF control groups.

The average diameter of the axons was also higher in GRAFT (4.15 ± 0.44) than in GRAFT+PE (2.93 ± 0.34) (p < 0.001), while remaining far from the values of the control group. Moreover, the G ratio (ratio between the diameter of the axon/fiber diameter, measured the degree of myelination fiber) of the GRAFT group (0.52 ± 0.03) had higher values than the GRAFT+PE group.

**Figure 4:**
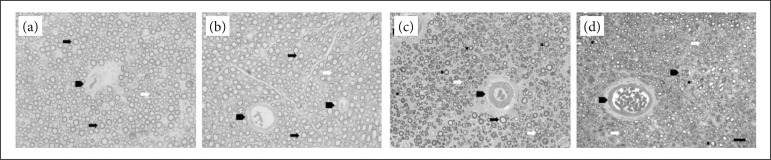
Thin sections photomicrograph of sciatic nerve, stained with toluidine blue. **(a)** SHAM; **(b)** SHAM+PE; **(c)** GRAFT; and **(d)** GRAFT+PE. Myelinated fiber of great caliber (black arrow). Myelinated fiber of small caliber (white arrow). Blood vessels (arrowhead). Wallerian degeneration (asterisk). Calibration bar: 20 μm, 400× magnification.

Two variables were not statistically significant between the GRAFT and GRAFT+PE groups: the thickness of the sheath (1.73 + 0.08 vs 1.56 + 0.08) and the density of myelinated fibers (53.62 + 12 14 vs 45.48 + 13.21), with p = 0.264 and 0.765, respectively (Table 2, Fig. 4).

**Table 2:** Results of morphometric variables among the groups.

	Groups	Mean	SD	95% CI	Minimum	Maximum	p-value
**Fiber diameter (**μ**m)**	SHAM	**11.53**	0.34	11.11–11.95	11.14	11.91	**p < 0.001** GRAFT vs. GRAFT+PE; **p < 0.001** SHAM/SHAM+PE vs GRAFT/GRAFT+PE; **p > 0.05** SHAM vs SHAM+PE
	GRAFT	7.76	0.27	7.41–8.10	7.47	8.16	
	GRAFT+PE	6.07	0.47	5.48–6.66	5.25	6.47	
	SHAM+PE	11.61	0.28	11.26–11.96	11.15	11.9	
**Axon Diametar (**μ**m)**	SHAM	6.22	0.23	5.92–6.51	5.99	6.56	**p < 0.001** GRAFT vs GRAFT+PE; **p < 0.001** SHAM/SHAM+PE vs GRAFT/GRAFT+PE; **p > 0.05** SHAM vs SHAM+PE
	GRAFT	4.15	0.44	3.60–4.71	3.71	4.69	
	GRAFT+PE	2.93	0.34	2.51–3.36	2.38	3.23	
	SHAM+PE	6.31	0.28	5.96–6.65	5.98	6.61	
**Sheat thickness (**μ**m)**	SHAM	2.65	0.17	2.44–2.87	2.54	2.96	**p = 0.264** GRAFT vs GRAFT+PE; **p < 0.001** SHAM/SHAM+PE vs GRAFT/GRAFT+PE; **p > 0.05** SHAM vs SHAM+PE
	GRAFT	1.73	0.08	1.63–1.83	1.61	1.82	
	GRAFT+PE	1.56	0.08	1.46–1.66	1.44	1.66	
	SHAM+PE	2.64	0.18	2.40–2.87	2.49	2.96	
**G ratio**	SHAM	0.53	0.01	0.51–0.55	0.5	0.55	**p = 0.044** GRAFT vs GRAFT+PE; **p < 0.001** SHAM/SHAM+PE vs GRAFT/GRAFT+PE; **p > 0.05** SHAM vs SHAM+PE
	GRAFT	0.52	0.03	0.49–0.56	0.5	0.58	
	GRAFT+PE	0.48	0.02	0.45–0.51	0.45	0.5	
	SHAM+PE	0.54	0.02	0.51–0.57	0.5	0.56	
**Total nerve area (**μ**m2)**	SHAM	229	19.81	204.40–252.59	205	260	**p = 0.005** GRAFT vs GRAFT+PE; **p < 0.001** SHAM/SHAM+PE vs GRAFT/GRAFT+PE; **p > 0.05** SHAM vs SHAM+PE
	GRAFT	144	46.55	86.19–201.80	100	195	
	GRAFT+PE	363	91.82	248.98–477.02	235	480	
	SHAM+PE	200	137.7	29.01–370.98	100	430	
**Density of mielinated fiber**	SHAM	41.68	5.39	34.97–48.38	34.6	48.5	**p = 0.026** GRAFT vs GRAFT+PE; **p < 0.001** SHAM/SHAM+PE vs GRAFT/GRAFT+PE; **p > 0.05** SHAM vs SHAM+PE
	GRAFT	53.62	12.14	38.54–68.69	37.5	67.1	
	GRAFT+PE	45.48	13.21	29.07–61.88	30.3	57.9	
	SHAM+PE	38.4	14.65	20.21–56.59	20	56.6	

### Spinal cord histology (motoneurons count)

When comparing the results of motoneuron count among the grafted groups, there was not any statistical difference, which was also the case when comparing the two control groups, SHAM and SHAM+PE.

In the SHAM and SHAM+PE groups, the number of motor neurons in the anterior horn of the spinal cord were 1,058 ± 165.7 and 1,001 ± 52.3, respectively. These values are significantly higher than those found in other groups: 834.5 ± 15.5 in GRAFT group (p < 0.001) and 833 ± 17.1 (p < 0.001) in the GRAFT+PE group (Table 3).

**Table 3:** Motor neuron counts with and without Abercrombie’s correction across the four groups.

		Mean	SD	95% CI	Minimum	Maximum	p-value
**Abercrombie**	SHAM	**687.88**	107.75	588.22–787.54	614.2	919.1	p > 0.05 (Graft vs. GRAFT+PE); p < 0.001 (Graft / GRAFT+PE vs. SHAM / SHAM+PE); p > 0.05 (SHAM vs. SHAM+PE)
	GRAFT	542.18	10.30	532.66–551.70	527.8	557.7	
	GRAFT+PE	541.45	11.14	532.87–550.02	527.8	560.3	
	SHAM+PE	650.65	33.99	608.43–692.86	605.8	691.0	
**Motoneurons**	SHAM	1058.29	165.76	904.97–1211.59	945	1,414	p > 0.05 (Graft vs. GRAFT+PE); p < 0.001 (Graft / GRAFT+PE vs. SHAM / SHAM+PE); p > 0.05 (SHAM vs. SHAM+PE)
	GRAFT	834.57	15.54	820.19–848.94	812	858	
	GRAFT+PE	833	17.15	819.81–846.18	812	862	
	SHAM+PE	1,001	52.30	936.05–1065.94	932	1,063	

## Discussion

The choice of the sciatic nerve in rats was due to the ease of microsurgical manipulation before its division^23^, histological resemblance to the human peripheral nerves^24^, and as a comparison with other studies. This study demonstrated that both GRAFT and GRAFT+PE groups when compared to SHAM control groups and SHAM+PE had worse results in all parameters (clinical, morphological, morphometric and histological).

When comparing only the clinical results of the GRAFT groups, they are similar to those found in the literature 60 days after neurorraphy (SFI60 = –73.4). Oliveira *et al*.^25^ obtained –83.46 with 5 mm graft, Jayakumar *et al*.^26^ –79.9 with 10 mm graft, and Sabongi *et al*.^27^ –76.4 with 8 mm graft. Komiyama *et al*.^28^, however, found better results with an SFI average value of –23.4 to 10 mm grafts.

Despite the review carried out by McGregor *et al*.^14^, with better functional results in most of the studies presented in exercises groups, the present study observed no clinical differences, measured by the SFI, when compared the group submitted to physical exercise with the control group (GRAFT vs GRAFT+PE). Although there is no significance, the final value (SFI90) of the group subjected to the protocol was better, perhaps these values are slightly improved by the rehabilitation function of physical activity protocol.

Ilha *et al*. 29, who also used SFI as a criterion for postsurgical assessment, noted an early improvement of SFI values in groups submitted to physical exercise, but these positive results may have shown up because the author caused Neuropraxia on a ciatic nerve. Asensio-Pinilla *et al*.^1^ used electrophysiological tests as a criterion after sectioning of the sciatic nerve in rats and their results (amplitude and wave latency values), over 60 days, were better in animals subjected to exercise when compared to the control. Other studies using clinical criteria, such as animal gait, in the evaluation^30^^,^ 31 yielded similar results, with better rates in the exercise groups. All these experiments used end-to-end neurorraphy, not nerve graft.

When evaluating the results of retrograde labeling with FG to check the connection of peripheral nerves in the central nervous system, the values of stained motor neurons in the anterior horn of the spinal cord in the GRAFT and GRAFT+PE groups showed no statistical difference, remaining well under the control groups (SHAM and SHAM+PE).

In morphologic and morphometric analysis, the increased fiber density in GRAFT groups is a commun find after nerve lesions. The number increases significantly up to 3 months, reaches a plateau between 6 to 9 months and than returns to control group values after a year^31^.

Comparing the GRAFT and GRAFT+PE, the first showed higher values, with a statistical significance, for fiber diameter (7.76 vs 6.07), diameter of the axon (4.15 vs 2.93) and density of myelinated fibers (53.62 vs 45.48). Different results were found by Ilha *et al*.^29^ and Asensio-Pinilla *et al*.^1^, who described best values in the group submitted to physical exercise. The G ratio was 0.54 in the SHAM group, realy close to the GRAFT group (0.528) and better than the GRAFT+PE group (0.48) (p < 0.05). Normally, this ratio is about 0.6, a value in which nerve conduction is more efficient. Values close to zero indicate axonal atrophy, while values close to one reflect fiber demyelination^32^^–^ 34. Despite these better results without exercise protocol no differences were found in clinical (SFI) and motoneuron analysis. This dissociation between the clinical and morphometric results may be related to the difficulty of clinical measurement with the SFI for small differences between values obtained in the microscopic evaluation of the nerve.

Several types of interventions were performed to achieve better functional results after neurorraphy, either with swimming^16^^,^ 34, treadmill, voluntary exercise^35^ and skill^36^ or resistence exercise^37^. A study comparing charge with exercises (treadmill) and no load (swimming) in these types of injury suggested that there is no difference between both^37^.

The choice on the type, frequency and intensity of exercise is still controversial. Some studies on a treadmill with high frequency and high-speed exercises report considerable^38^^–^ 40 muscle damage as well as inhibition of the sides and terminal buds and with worse results than the control group^41^.

The beginning three weeks were based on other studies, considering a reduction in post surgical inflammation, healing of the wound and applicability of the protocol to humans^42^. The early application of the training after peripheral nerve injury is considered to be a prognostic factor for good results. Most studies had positive results with this intervention had their protocols initiated between 1 day and 3 weeks after surgery^1^^,^ 29 ^,^ 38 ^,^ 41 ^,^ 43. Some other studied benefits described of the early initiation of physical activity in the postoperative period are the improvement of neuropathic pain after these injuries^44^.

It is difficult to compare directly the results of this study with those published in the literature since the surgical technique (size of the graft), the beginning of the protocol time, intensity, frequency and methods of measurement, in most cases, are different.

## Conclusion

This experimental model shows that this protocol of aerobic treadmill exercises did not modify nerve regeneration after sciatic nerve injury and repair with nerve graft. Further studies are needed to evaluate methods to accelerate and improve functional results after neurorraphy peripheral nerve graft.
